# The novel *Arabidopsis thaliana* *svt2* suppressor of the ascorbic acid-deficient mutant *vtc1-1* exhibits phenotypic and genotypic instability

**DOI:** 10.12688/f1000research.2-6.v1

**Published:** 2013-01-10

**Authors:** Chase F Kempinski, Samuel V Crowell, Caleb Smeeth, Carina Barth

**Affiliations:** 1 Department of Biology, West Virginia University, Morgantown, 26506, USA; 2 Department of Plant and Soil Sciences, University of Kentucky, Lexington, 40546, USA; 3 Department of Plant Biology, Cornell University, Ithaca, 14853, USA; 4 ACTION-Housing Inc., Pittsburgh, 15219, USA; 5 ConRuhr North America, New York, 10017, USA

## Abstract

Ascorbic acid is a potent antioxidant that detoxifies reactive oxygen species when plants are exposed to unfavorable environmental conditions. In addition to its antioxidant properties, ascorbic acid and its biosynthetic precursors fulfill a variety of other physiological and molecular functions. A mutation in the ascorbic acid biosynthesis gene *VTC1*, which encodes GDP-mannose pyrophosphorylase, results in conditional root growth inhibition in the presence of ammonium. To isolate suppressors of *vtc1-1*, which is in the *Arabidopsis* Columbia-0 background, seeds of the mutant were subjected to ethyl methanesulfonate mutagenesis. A suppressor mutant of *vtc1-1* 2, *svt2*, with wild-type levels of ascorbic acid and root growth similar to the wild type in the presence of ammonium was isolated. Interestingly, *svt2* has *Arabidopsis* Landsberg *erecta* features, although *svt2* is delayed in flowering and has an enlarged morphology. Moreover, the *svt2 *genotype shares similarities with L *er* polymorphism markers and sequences, despite the fact that the mutant derived from mutagenesis of Col-0 *vtc1-1* seed. We provide evidence that *svt2* is not an artifact of the experiment, a contamination of L *er *seed, or a result of outcrossing of the *svt2* mutant with L *er* pollen. Instead, our results show that *svt2* exhibits transgenerational genotypic and phenotypic instability, which is manifested in a fraction of *svt2* progeny, producing revertants that have Col-like phenotypic and genotypic characteristics. Some of those Col-like revertants then revert back to *svt2*-like plants in the subsequent generation. Our findings have important implications for undiscovered phenomena in transmitting genetic information in addition to the Mendelian laws of inheritance. Our results suggest that stress can trigger a genome restoration mechanism that could be advantageous for plants to survive environmental changes for which the ancestral genes were better adapted.

## Introduction

l-ascorbic acid (AA, vitamin C) is an important antioxidant with multiple functions in many species. It serves as a scavenger of reactive oxygen species generated under adverse environmental conditions. However, AA also influences flowering time and senescence ^1– 3^, pathogen disease resistance ^2, 4^, the biosynthesis of various plant hormones ^5– 7^, and root development ^8– 11^. This suggests that AA and some of its intermediates have functions in addition to its antioxidant properties.

Ascorbic acid biosynthesis in plants occurs predominantly through the d-mannose/ l-galactose pathway ^12, 13^. Given the multifaceted functions of AA in plants, there is a need to advance our understanding of how plants regulate the biosynthesis and accumulation of AA. *Arabidopsis thaliana* mutants deficient in AA have provided important insights into the breadth of molecular and physiological functions of AA. One of the *Arabidopsis* mutants, *vtc1-1*, contains a defect in the AA biosynthetic enzyme GDP-mannose pyrophosphorylase. The mutant was originally generated by ethyl methanesulfonate (EMS) mutagenesis of Col-0 wild-type seed ^14^. The *vtc1-1* mutant contains a point mutation in amino acid 22 that converts a conserved proline into a serine ^15^. The *VTC1* gene has recently been shown to be a determinant of ammonium sensitivity in plants. In the presence of ammonium, *vtc1-1* mutants exhibit strongly reduced root growth in comparison to the wild type, a phenomenon that is independent of AA deficiency ^8– 11^. To better understand the mechanism through which VTC1 mediates conditional ammonium sensitivity, it is important to identify regulatory partners of VTC1. To accomplish this, we undertook a suppressor mutagenesis approach of *vtc1-1* homozygous mutant seed in the hope of identifying *vtc1-1* suppressor mutants that could then be isolated and studied.

One of the suppressor mutants isolated in the M _0_ generation, *svt2* ( *suppressor of vtc1-1 2*), contained wild-type AA levels and developed roots similar to the wild type in the presence of ammonium. However, while characterizing the mutant genotypically, we observed that it lost the original *vtc1-1* mutation (i.e., *svt2* contained the homozygous wild-type allele). Furthermore, we determined that *svt2*, although generated through EMS mutagenesis of Col-0 *vtc1-1* mutant seed, was phenotypically and genotypically similar to L *er*. Intriguingly, a small percentage of *svt2* M _1_ plants produced offspring that have phenotypic and genotypic similarities to Col in the M _2_ generation. Even more remarkably, a small percentage of Col-like revertants in the M _2_ generation produced progeny that exhibited phenotypic and genotypic *svt2* characteristics again in the M _3_ generation.

Phenotypic instability of *Arabidopsis* alleles affecting a disease resistance gene cluster has recently been reported ^16^. In their work, Yi and Richards described that exposure to EMS or through the generation of different F _1_ hybrids induced phenotypic instability in the *bal* and *cpr1* mutant alleles. The authors later proposed that the high phenotypic instability is caused by a genetic mechanism ^17^.

The presented study focuses on describing and characterizing the *Arabidopsis svt2* suppressor mutant and its phenotypic and genotypic behavior. After illustrating the phenotypic features of *svt2*, we investigate transgenerational changes in the phenome and genome of *svt2* and provide evidence that *svt2* is a true mutant and not the result of an experimental artifact or contamination. Finally, we discuss our experimental findings in respect to the *vtc1-1* mutant background and other reports that previously described similar phenomena of genome instability and restoration, and we briefly speculate on possible mechanisms of phenome and genome instability in *svt2*.

## Materials and methods

### Plant material and growth conditions

*Arabidopsis thaliana* Col-0 wild type and the previously described *vtc1-1* mutant ^14^ (in the Col-0 background) were kindly provided by Patricia Conklin (SUNY Cortland, NY, USA). L *er*-0 wild-type seed were obtained from The Arabidopsis Biological Resource Center ( http://www.arabidopsis.org). Plants were grown in Metromix 360 potting soil at 23°C at both day and night with a 16-hour photoperiod at 160 μmol photons m ^-2^ s ^-2^ (fluorescent bulbs).

For assessment of root growth, seed of the wild types and mutant lines were surface-sterilized (see below) and grown on basal full strength 1× Murashige and Skoog (MS) medium without vitamins (Cat.# MSP01, Caisson Laboratories, Inc., North Logan, UT), containing 1% Phytoblend (Cat.# PTP01, Caisson Laboratories) in omni trays (Fisher Scientific, Pittsburgh, PA) as described ^11^. Sucrose was omitted from the tissue culture medium. The pH of the medium was adjusted with KOH to 5.7. Trays were sealed with two layers of 3M micropore tape (Fisher Scientific), put in vertical orientation, and placed in the growth chamber under long days (16 h light, 8 h dark) at 23°C day and night, and 160 µmol photons m ^-2^ s ^-1^ in a growth chamber (Percival Scientific, Inc., Perry, IA). Each plate contained wild-type and mutant seed. Primary root length was measured in seven-day-old seedlings using a ruler.

To assess AA content in leaf tissue, seeds of wild type and mutants were randomly sown on MetroMix 360 soil (BFG supplies Co., Burton, OH) in the same flat under the growth conditions described above. When plants were three weeks old, whole rosettes were harvested for the AA assay.

### Seed-surface sterilization

Seeds were soaked for 1 min in 50% ethanol, followed by washing the seeds in 50% bleach plus 0.01% sodium dodecyl sulphate for 6 min. Finally, seeds were rinsed six times with sterile water and stored in 0.1% sterile Phytoblend agar for 2 d at 4°C ^18^.

### Ethyl methanesulfonate mutagenesis

**Figure 1.**
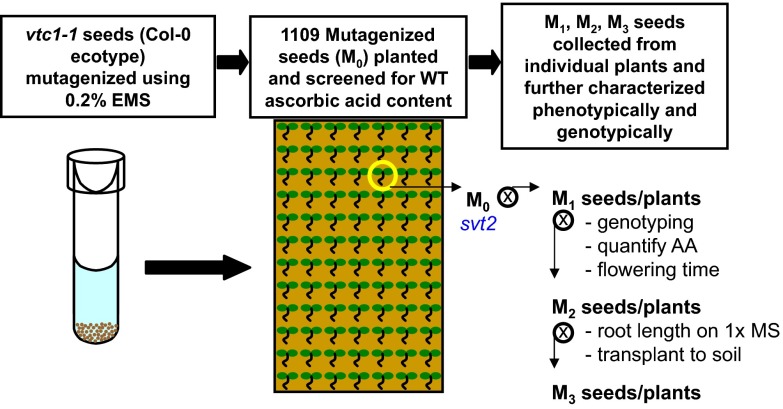
Isolation of *svt2*. To isolate *vtc1-1* suppressor mutants, homozygous *vtc1-1* seed (in the Col-0 genetic background) were exposed to chemical mutagenesis using ethyl methanesulfonate (EMS). Over 1000 mutagenized seed (M _0_) were planted on soil and screened for wild-type levels of ascorbic acid. The only mutant isolated in the M _0_ generation containing recovered ascorbic acid levels was *svt2*. The mutant was allowed to self-fertilize and was characterized phenotypically and genotypically in subsequent generations.

Seeds of homozygous *vtc1-1 Arabidopsis thaliana* (Col-0 background) were mutagenized with 0.2% ethyl methanesulfonate as described ( Figure 1; ^18^). Approximately, 1200 M _0_ seed were stratified for 4 days at 4°C in 0.1% agar, sown on MetroMix soil and grown as above. Plants were screened for wild-type AA levels using the nitroblue tetrazolium assay ^19^. Additional suppressor mutants were isolated by pooling seeds generated from M _1_ plants. Putative mutants were isolated and allowed to self-pollinate to obtain seed.

### Pollen grain analysis and microscopy

Pollen was taken from 4.5-week-old flowering plants of Col-0 and L *er* wild type and *vtc1-1* and *svt2* M _2_ mutants, mounted in glycerol, and photographed using bright field settings on a Nikon E800 microscope equipped with a CoolSNAP cf CCD camera (Photometrics, Tuscon, AZ, USA).

### Genomic DNA isolation

Genomic DNA was isolated from rosette leaves following a previously described protocol ^3^. In case of genomic DNA isolation from *vtc1-1* seeds, a small amount of dried seeds was crushed and the extraction procedure described previously ^3^ was followed. Primers for the *VTC1* gene and for the Insertion/Deletion (InDel) polymorphisms were designed using sequence data available on The Arabidopsis Information Resource (TAIR) database ( http://www.arabidopsis.org). Polymerase chain reaction (PCR) was used to amplify fragments of the *VTC1* gene for sequencing and to assess InDel polymorphisms. Sequences of primers used for sequencing and InDel analysis are summarized in Table 1. PCR reactions were run on 1.0% agarose gels stained with ethidium bromide.

**Table 1.** Forward (F) and reverse (R) sequences of primers used in analyzing the *VTC1* gene and for amplifying five Col/L *er* Insertion/Deletion (InDel) polymorphisms.

Primer Name	5´ 3´
*VTC1* G1 F	AAA AAT TCG TTC TAG ATG GAT GCT
*VTC1* G1 R	ATG GCT GTA AAT TGG AAG AGA T
*VTC1* G2 F	GAA CCC TTG TCT CTA AAA TA
*VTC1* G2 R	CAA ATC CCA TAA TCT GTT CC
*VTC1* G3 F	CAA TTT TGC TTA CTT CTC T
*VTC1* G3 R	TGG ATG CAA CCG ACA CAA AAC AAT
*VTC1* G4 F	ACA TTT TTA GCA GCT GGT ATT GAG
*VTC1* G4 R	AGG TAA GAA CTG GCA GAC TAA AG
*VTC1* G5 F	TCG CTT GAG ACC ATT GAC T
*VTC1* G5 R	GAG GCT TCC CCA CCG TGA GAT TTG
*VTC1* G6 F	CAA GCT GGA AAT CAA AAT CAC T
*VTC1* G6 R	GCG CTG CTG CAA TCT TAG G
*VTC1* G7 F	ACA AAT CTC ACG GTG GGG AAG C
*VTC1* G7 R	TGG TTA ATT TGG CAG GAG A
*VTC1* G8 F	CAA GGG CTC TAT GCT ATG GTG
*VTC1* G8 R	GCG TTT TGA TTG ATG CTT ATT C
*VTC1* G9 F	GCG TGT ATC TCG AGC AGT ATC AT
*VTC1* G9 R	GTG GAG GGA AGT TAA GGG TAT TTT
InDel 1 450919 F	ATC GGT TTG TAA TCT CTG TCC A
InDel 1 450919 R	TAT GCG TTC CCA AAT TTG TTA TCT C
Indel 2 451470 F	GGA GAC CCA AAC TGC TAT TAC A
Indel 2 451470 R	AAC CGC CTC CAT TTG CAC CTT ATC
Indel 3 469762 F	GTC ACC GAG TTT TGC TTT GTT CAT
Indel 3 469762 R	CTC GTT TCT TTT CTG GGC TTG TAG
Indel 4 449053 F	GAA AGA AAG CAG CGA AAG ACA
Indel 4 449053 R	GCC CAT GCC CAT ACA CTG A
Indel 5 455100 F	ACT TGC TTA ATC GTT TCT TTG TA
Indel 5 455100 R	GCC CAC TCG TAT TCG CTT AG

### Gene copy analysis using qPCR

Quantitative PCR reactions were set up to measure gene copy number using 2.5 pmole gene-specific primers, 300 ng of genomic DNA diluted in DNase/RNase free water, and iQ SYBR Green supermix (Bio-Rad, Hercules, CA, USA) for a total volume of 10 μL. Reactions without template were used as negative controls. Each single copy reaction was set up in triplicate and run in a Bio-Rad iCycler for 40 cycles. Threshold cycles (C _T_) were calculated using iQ software (Bio-Rad).

Primer efficiencies (E) were calculated using cDNAs synthesized from RNA isolated from Col-0 plants as previously described ^11^. cDNA samples were serially diluted across three orders of magnitude. Serial dilutions were amplified in triplicate using the same protocol as for the copy number experiment. The C _T_s of each triplicate were averaged and plotted against the dilution factor. A linear trend was fitted to the data and the slope of this trend was used to calculate E for each primer with the formula: E=10 ^(1/-slope)^.

Copy number of *VTC1* (AT2G39770) was calculated using the formula: Reported Quantity (RQ) = 1/E ^CT^ normalized to the RQ of a known single copy gene ( *PAD4*, AT3G52430; ^20, 21^). *VTC1* RQ was calculated from the average *VTC1* RQ of three biological replicates per genotype and was normalized to the average RQ of *PAD4* from three replicates of each respective genotype, all run in the same reaction plate.

### Sequencing analysis

PCR products were purified using the Qiagen Miniprep Kit. Dye-terminator based DNA sequencing was performed at the Genomics Facility in the Department of Biology at West Virginia University. Sequence alignments were performed using the BioEdit program ( http://www.mbio.ncsu.edu/bioedit/bioedit.html).

### Ascorbic acid quantification

To screen mutants, AA levels were analyzed qualitatively in small pieces of two-week-old rosette leaves using the nitroblue tetrazolium assay previously described ^19^. The AA content was determined in whole rosettes of three-week-old plants using the iron reduction assay ^4^.

### Statistical analysis

Experiments were performed at least three times. Figures represent individual experiments. Data were expressed as mean values ± SE. *P* values were determined by Student’s *t* test analysis.

## Results

### Isolation of *svt2*

**Figure 2.**
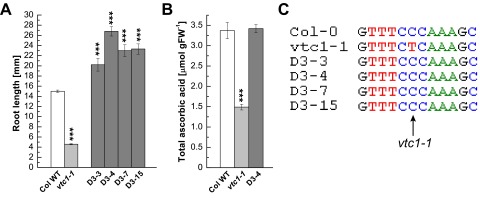
Phenotypic and genotypic characterization of additional *vtc1-1* suppressor mutants. ( **A**) Root length in seven-day-old seedlings grown on 1× MS. Bars represent means ± SE of 18–73 individuals. Since D3-4 homogenously produced long roots, all individuals were included in the calculations. As D3-3, D3-7, and D3-15 developed long and short roots in an approximate 3:1 ratio, only individual seedlings that produced long roots were included in the calculations. ( **B**) Total ascorbic acid content per gram fresh weight in whole rosettes of three-week-old plants. Bars represent means ± SE of three (Col-0 and *vtc1-1*) or 24 individual replicates. *** *P* < 0.001 by Student’s *t*-test indicates significant differences in comparison to the Col-0 wild type. ( **C**) Sequences of the Col-0 wild type, the *vtc1-1* mutant and four suppressor mutants. The arrow points to the *vtc1-1* mutation, a conversion of cytosine to a thymine.

Our laboratory is interested in understanding how the *VTC1* gene, which is essential for the biosynthesis of GDP-mannose and AA, is regulated. This would help deciphering the pleiotropic phenotypes displayed by *vtc1-1*, including its hypersensitivity to ammonium ^8– 11^. We employed a gene suppressor analysis with the goal of identifying novel genes that interact or regulate *VTC1*. Seed of the *vtc1-1* mutant, which is in the Col-0 genetic background ^14^, were subjected to chemical mutagenesis using EMS ^18^. One thousand and one hundred mutagenized *vtc1-1* seeds (M _0_ generation) were planted onto soil and screened for recovered (wild-type) leaf AA content using the qualitative nitroblue tetrazolium test ^19^. One of the mutants exhibited wild-type AA levels in the M _0_ generation. This mutant was named *svt2* ( *suppressor of vtc1-1 2*), isolated, and further characterized. The mutant was allowed to self-fertilize and seeds from the plant were collected (M _1_ generation) ( Figure 1). Note that we isolated additional suppressor mutants by pooling M _2_ seed and by screening for long roots on 1× Murashige and Skoog (MS) medium containing ammonium. Six suppressor mutants were identified among 2000 plants. M _3_ seed were collected and screened for long roots again to test for segregation. M _4_ progeny of one line had all long roots, whereas the other five lines segregated in a ratio of three plants producing long roots, and one plant having short roots. Figure 2 summarizes data of four of these suppressor mutants, with D3–4 homogenously producing long roots, whereas D3–3, D3–7, and D3–15 developed long and short roots in a 3:1 ratio. As is illustrated in Figure 2A, these suppressor mutants developed roots that were significantly longer than those of the Col-0 wild type. Analysis of the total AA content revealed that the suppressor D3–4 had an AA content comparable to the Col-0 wild type, whereas that of *vtc1-1* was only approximately 40% of that of the wild type ( Figure 2B) ^14, 15^. Finally, sequence analysis of these four suppressor mutants demonstrated a lack of the *vtc1-1* mutation ( Figure 2C). Except for the assessments described above, these suppressor mutants were not yet characterized further.

Root lengths of Col-0 WT, vtc1-1, and D3 suppressor mutants (mm) Root length in seven-day-old seedlings grown on 1x MS

Total ascorbic acid of Col-0 WT, vtc1-1, and D3 suppressor mutants (μmol gFW-1) Total ascorbic acid content per gram fresh weight in whole rosettes of three-week-old plants

### *svt2* has similarities with the L *er* phenotype, but has also phenotypic characteristics that are distinct from L *er*

**Figure 3.**
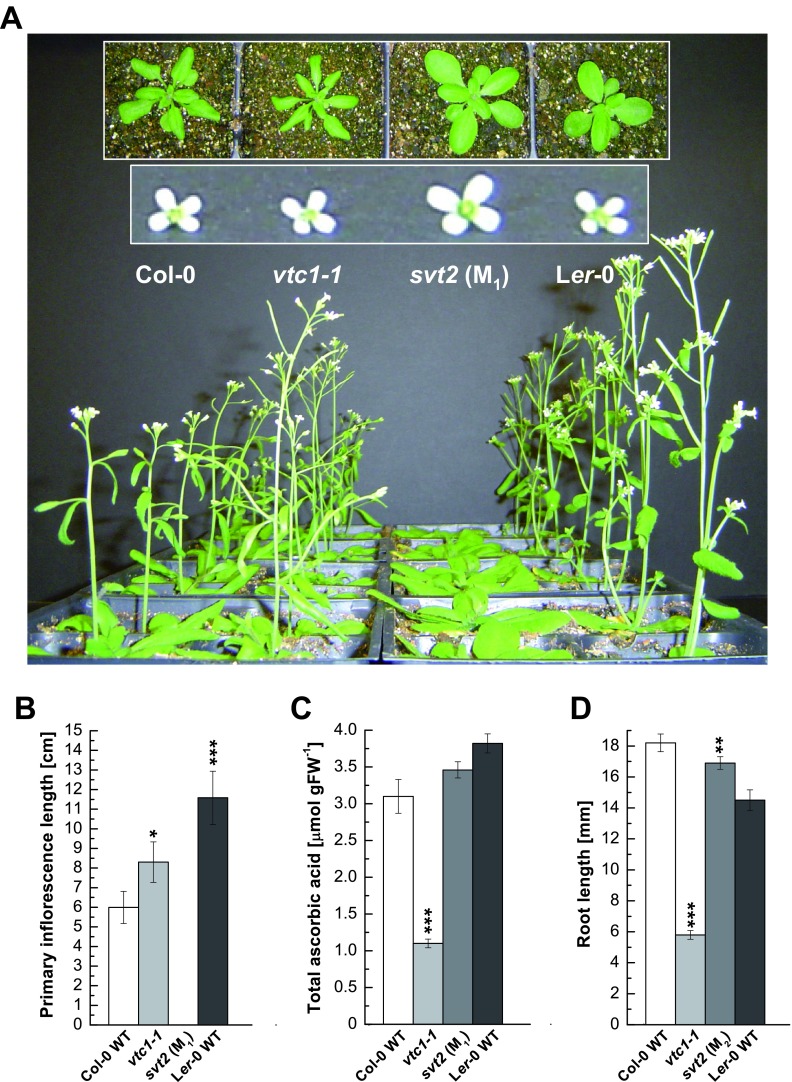
Phenotypic characterization of *svt2*. ( **A**) Flowering phenotype of four-week-old Col-0 wild type, the *vtc1-1* and *svt2* mutants and the L *er*-0 wild type. Insets show rosette phenotypes of the four genotypes when plants were three weeks old and the flower phenotype of six-week-old plants, respectively. ( **B**) Primary inflorescence length when plants were four weeks old. Bars represent means ± SE of eight individual replicates. ( **C**) Total ascorbic acid content per gram fresh weight in whole rosettes of three-week-old plants. Bars represent means ± SE of three individual replicates. ( **D**) Root length in seven-day-old seedlings grown on 1× MS. Bars represent means ± SE of 30–90 individuals. * *P* < 0.05, ** *P* < 0.01, *** *P* < 0.001 by Student’s *t*-test indicate significant differences in comparison to Col-0 and L *er*-0 wild type, respectively.

The first observation we made when characterizing *svt2* M _1_ plants was that *svt2* exhibited a phenotype reminiscent of the L *er* ecotype with the characteristic round leaves and erect morphology when compared to Col ( Figure 3A). However, *svt2* also had features that were distinct from the L *er* phenotype, including overall enlarged vegetative and reproductive morphology (insets of rosettes and flowers in Figure 3A). In addition, *svt2* was strongly delayed in flowering compared to the Col-0 and L *er*-0 wild types and the *vtc1-1* mutant ( Figure 3A, 3B). Primary inflorescences in four-week-old plants were 1.4-times significantly longer in the *vtc1-1* mutant and approximately twice as long in the L *er*-0 wild type compared to the Col-0 wild type. In *svt2* mutant plants, however, buds of primary inflorescences only began to emerge when plants were four weeks old ( Figure 3A, 3B). The flowering data are consistent with previous reports, with L *er*-0 wild type entering the reproductive phase before Col-0 wild type. An early flowering phenotype of *vtc1-1* has been reported previously ^3^.

Primary inflorescence length of Col-0 WT, vtc1-1, svt2 (M1) and Ler-0 WT (cm) Primary inflorescence length when plants were four weeks old

Total ascorbic acid of Col-0 WT, vtc1-1, svt2 (M1) and Ler-0 WT (μmol gFW-1) Total ascorbic acid content per gram fresh weight in whole rosettes of three-week-old plants

Root lengths of Col-0 WT, vtc1-1, svt2 (M1) and Ler-0 WT (mm) Root length in seven-day-old seedlings grown on 1x MS

The AA content in *svt2* was similar to levels quantified in Col-0 and L *er*-0 wild types, whereas *vtc1-1* contained approximately 30% of the AA content as expected ^14, 15^ ( Figure 3C). Finally, we investigated whether *svt2* also exhibits recovered root development in the presence of ammonium by growing the four genotypes in full strength 1× MS medium. The *vtc1-1* mutant is conditionally hypersensitive to ammonium ^8, 9, 11^. Figure 3D illustrates that root length in *svt2* was the same as in Col-0 wild type, whereas root development was strongly inhibited in *vtc1-1* as expected.

**Figure 4.**
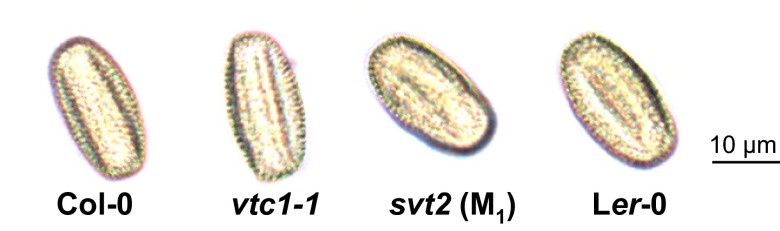
Pollen phenotype of Col-0 wild type, the *vtc1-1* and *svt2* mutants and the L *er*-0 wild type. Light images were taken when plants were 4.5 weeks old. Scale bar represents 10 µm.

The enlarged morphology of *svt2* raises the question as to whether *svt2* is polyploid. In order to test this, we assessed the size of pollen grains from the Col-0 and L *er*-0 wild-types and *vtc1-1* and *svt2* mutants. As is shown in Figure 4, pollen grains of the four genotypes are similar in size. In addition, using qPCR, we determined the number of *VTC1* gene copies in the four genotypes. Our results revealed that *VTC1* is present as a single copy gene in both the Col-0 and L *er*-0 wild types and in the *vtc1-1* and *svt2* mutants ( Table 2). Although an extensive chromosome analysis has not yet been performed in *svt2*, our results suggest that the mutant does not contain additional sets of chromosomes.

**Table 2.** Quantitative PCR to verify that *VTC1* is a single copy gene in Col-0 and L *er*-0 wild types and *vtc1-1* and *svt2* mutants. Quantitative PCR was performed as described in Materials and Methods. The *PAD4* gene is a known single copy gene. Therefore, an RQ/RQ ratio of approximately 1 indicates that *VTC1* is present in similar quantity as *PAD4*, and therefore a single-copy gene.

	RQ/RQ	
	*VTC1/PAD4*	
**Col-0 WT**	0.3796	**VTC1** **E=1.8**
***vtc1-1***	0.5843	
***svt2***	0.5504	
**L *er*-0 WT**	0.6329	
**Col-0**	0.3153	**VTC1** **E=2**
***vtc1-1***	0.5292	
***svt2***	0.4946	
**L *er*-0 WT**	0.5807	

Quantitative PCR to verify that VTC1 is a single copy gene in Col-0 and Ler-0 wild types and vtc1-1 and svt2 mutants Quantitative PCR was performed as described in Materials and Methods. The PAD4 gene is a known single copy gene. Therefore, an RQ/RQ ratio of approximately 1 indicates that VTC1 is present in similar quantity as PAD4, and therefore a single-copy gene

Taken together, based on the phenotypic observations, our data suggest that *svt2* represents a novel *vtc1-1* suppressor mutant with recovered AA content and root development. Next, we characterized *svt2* genotypically in order to determine whether *svt2* represents an intragenic or extragenic suppressor.

### *svt2* shares genome similarity with L *er*

**Figure 5.**
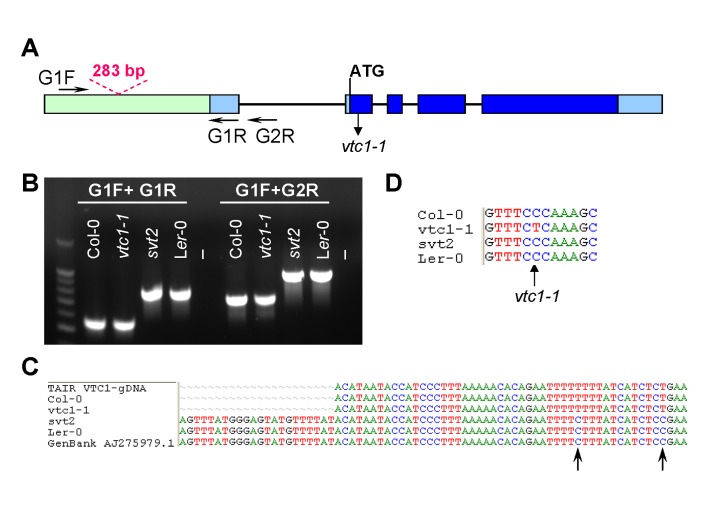
Genotypic characterization of *svt2*. ( **A**) *VTC1* Col-0 gene model. Light green box indicates *VTC1* gene promoter region, light blue rectangles indicate 5´ and 3´ UTRs, dark blue rectangles indicate exons, and lines indicate introns. Shown is the location of the *vtc1-1* mutation within the first exon, primer locations, and polymorphism insertion of 283 bp in L *er*-0 *VTC1*. ( **B**) PCR amplification of the *VTC1* promoter region in the Col-0 wild type, *vtc1-1* and *svt2* mutants and L *er*-0 wild type. (-) indicates negative control, no DNA. ( **C**) Partial sequence alignment of the *VTC1* promoter region from the TAIR database (Col-0), sequenced Col-0 wild type, *vtc1-1* and *svt2* mutants, sequenced L *er*-0 wild type and the L *er*-0 sequence obtained from GenBank. The alignment shows the sequence insertion in the *svt2* mutant, the L *er*-0 wild type and the GenBank sequence. Arrows indicate single nucleotide polymorphisms between the L *er*-0 and Col-0 sequence. ( **D**) Point mutation in *vtc1-1*, a conversion from a cytosine to a thymine.

**Figure 6.**
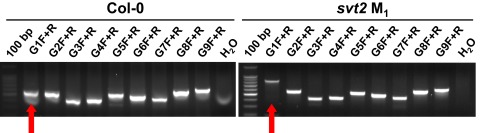
Molecular characterization of *svt2*. Amplification of the *VTC1* gene including ~500 bp of the promoter region using a series of nine, overlapping primers (G1F+R through G9F+R) in both Col-0 wild type and *svt2* M _1_ mutant genomic DNA. The last lane in each gel contained a negative control (water instead of DNA). Red arrows indicate the different sized PCR products using the same primer set.

To determine whether *svt2* represents an intragenic suppressor, i.e., to test whether the suppressor mutation is present within the *VTC1* gene, we designed nine overlapping PCR primer sets spanning the entire *VTC1* gene and approximately 500 bp of the promoter region directly upstream of the first base in the 5’ UTR ( Table 1, Figure 5A). PCR products were generated from genomic DNA extracted from Col-0 and L *er*-0 wild types, and *vtc1-1* and *svt2* mutants. In eight of the nine primer pairs covering the entire *VTC1* gene, the PCR products generated using *svt2* genomic DNA had the same electrophoretic mobility as those generated using Col-0 wild-type genomic DNA. However, this was not the case for the first primer set. The G1F/G1R primer set, used to amplify the *VTC1* promoter region, generated a larger PCR product in *svt2* than in the wild type ( Figure 5B, Figure 6). The PCR product in the wild type was 567 bp, whereas that in *svt2* had a size of approximately 850 bp, suggesting that *svt2* contained an approximately 300 bp insertion in this region. We repeated the PCR analysis of the *VTC1* promoter region using the G1F-G1R and the G1F-G2R primer sets that should generate a PCR product of 567 bp and 751 bp, respectively ( Figure 5A). The expected size was obtained for the Col-0 wild type and the *vtc1-1* mutant. However, approximately 300 bp larger PCR products were detected in the *svt2* mutant and the L *er*-0 wild type ( Figure 5B), suggesting a L *er* insertion polymorphism. Thus, these data imply that *svt2* shares both phenotypic and genotypic similarities with L *er*.

PCR amplification of the VTC1 promoter region in the Col-0 wild type, vtc1-1 and svt2 mutants Additional raw data of PCR amplification of the VTC1 promoter region using the VTC1 G1F and VTC1 G2R primers in genomic DNA isolated from individual Col-0 wild type, svt2, and vtc1-1 plants (N=8 for each). Positive and negative controls are indicated as (+) and (-), respectively.

**Figure 7.**
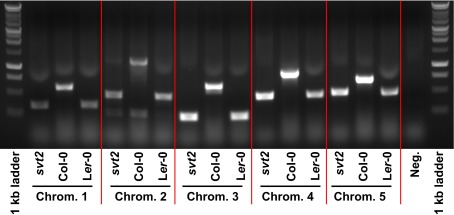
Insertion/Deletion polymorphism analysis in *svt2*, Col-0 and L *er*-0. Primers were designed for five randomly selected InDel polymorphisms across the five *Arabidopsis* chromosomes. The polymorphisms represent insertions in Col-0 and deletions in L *er*.

We therefore assessed five additional Insertion/Deletion (InDel) polymorphisms randomly chosen across the five *Arabidopsis* chromosomes ( Table 1) in *svt2* compared to the Col-0 and L *er*-0 wild types and sequenced the entire *VTC1* gene and the promoter region tested. Our data show that the PCR products generated for those five InDels using *svt2* genomic DNA had the same electrophoretic mobility as those produced from L *er*-0 genomic DNA ( Figure 7). Moreover, sequence analysis of the *VTC1* gene and promoter region revealed that *svt2* contained a 283 bp insertion in the *VTC1* promoter ( Figure 5C). The insertion is highlighted in gray in Figure S1. Note additional single nucleotide polymorphisms as indicated by upright arrows in Figure 5C and Figure S1. When we aligned the *VTC1* gene sequence obtained from *svt2* with that of the *vtc1-1* mutant, the *VTC1* Col-0 gene sequence deposited in the TAIR database, and the *VTC1* L *er* GenBank sequence, the *VTC1* gene sequence in *svt2* shared similarities with L *er* (upright arrows in Figure 5C, Figure S1) and Col (arrows pointing down in Figure S1). However, note that there are sequences that are unique to *svt2* and are not shared between Col, *vtc1-1* or L *er* (arrowheads in Figure S1). Finally, note the overlap in sequences between Col, *vtc1-1*, *svt2* and L *er* on the 5´ end of the sequence flanking the insertion (at approximately base pair 1990); see left-facing horizontal black arrow in Figure S1 compared to the sequence flanking the 3´ end of the DNA sequence insertion (starting at base pair 2273); see right-facing horizontal black arrow in Figure S1.

Finally, most intragenic suppressor mutants still contain the original mutation in addition to the suppressor mutation. Therefore, we expected that the *vtc1-1* mutation is still present in *svt2*. However, our sequencing analysis demonstrated that *svt2* did not contain the *vtc1-1* mutation anymore and that the mutation reverted back to the homozygous wild-type allele ( Figures 5D; green shading in Figure S1).

In summary, our data demonstrate that *svt2* shares DNA sequence similarity with Col and L *er*, but also contains DNA sequences that are unique to this mutant. This is particularly remarkable because *svt2* was generated in the *vtc1-1* Col-0 background. Also, *svt2* did not contain the original *vtc1-1* mutation anymore. Although our data already argue against *svt2* being a result of an artifact of the experiment or a contamination with L *er*, we analyzed subsequent *svt2* generations and discovered additional characteristics that are unique to *svt2*.

### *svt2* exhibits phenotypic and genotypic instability

Our initial observations revealed that approximately 10% of *svt2* M _2_ plants displayed a Col-like phenotype. Therefore, we planted *svt2* M _1_, M _2_, and M _3_ progeny to check whether this result could be repeated and to determine segregation ratios ( Table 3). Additionally, we investigated whether *svt2* progeny that were phenotypically Col-like revertants would produce *svt2* (L *er*-like) offspring in the next generation.

**Table 3.** Summary of revertant data. The table summarizes the number of plants screened in each of three *svt2* generations (M _1_, M _2_ and M _3_), screens of revertant progeny from Col-like revertants (A8, G7, K1), and the revertant progeny of a L *er*-like line (K1 Col R *svt2* R). The percent reversion is shown in the last column. Although the number of progeny plants tested is relatively large, some lines did not give rise to revertant progeny. R denotes revertant. *indicates mutant plants that were also analyzed genotypically (see Table 4).

Experiment	Generation	Total # of plants	# of phenotypic revertants	% reversion
**1**	*svt2* M _1_	63	0	0
	*svt2* M _2_, 3 of 7 revertants tested further: *svt2* A8 Col R M _3_ *svt2* G7 Col R M _3_ *svt2* K1 Col R M _3_* *svt2* K1 Col R *svt2* R M _4_*	78 64 64 63 96	7 (Col phenotype) 0 0 1 ( *svt2* phenotype) 0	8.97 0 0 1.58 0
	*svt2* M _3_	96	0	0
**2**	*svt2* M _1_	96	1 (Col phenotype)	1.04
	*svt2* M _2_, 2 of 5 revertants tested further: *svt2* Col R1 M _3_* *svt2* Col R4 M _3_*	62 88 96	5 (Col phenotype) 20 ( *svt2* phenotype) 0	8.06 22.73 0
**3**	*svt2* M _2_	96	10 (Col phenotype)	10.42

**Figure 8.**
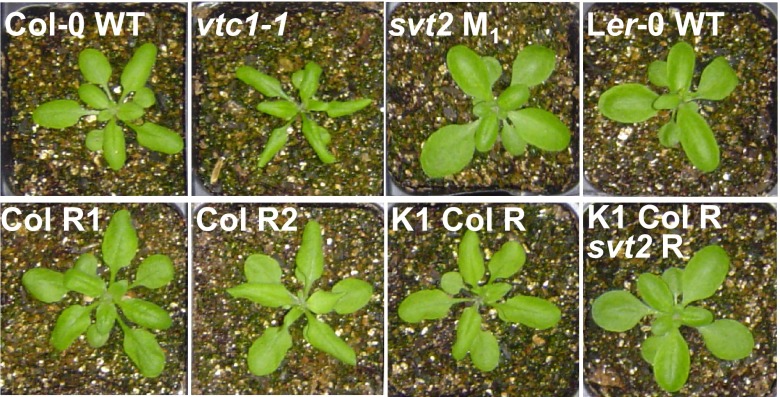
Phenotype of *svt2* revertants. Plants were three weeks old when photographs were taken. Top row represents controls, Col-0 wild type, *vtc1-1* and *svt2* mutants, and L *er*-0 wild type. Bottom row represents three Col-like revertants, *svt2* Col R1 M _3_, *svt2* Col R2 M _3_, *svt2* K1 Col R M _3_, and a double revertant, *svt2* K1 Col R *svt2* R M _4_. R stands for revertant.

As summarized in Table 3, revertants could only be detected when a relatively large population was planted. In the *svt2* M _1_ generation, only 1% of Col-like revertants were detected. In contrast, 8–10% of *svt2* M _2_ plants displayed a Col-like phenotype, whereas no revertants were detected in the *svt2* M _3_ generation. These Col-like revertants were isolated and seeds were collected from individual plants and the phenotype of the progeny in the M _3_ generation was assessed in some examples. In most cases, reversion appeared to be stable, i.e., once *svt2* plants reverted, displaying a Col-like phenotype in the M _2_ generation, their M _3_ progeny continued to appear as Col-like plants. This was the case for the M _3_ progeny of the A8 and G7 plants listed in Table 3. However, out of 63 progeny from the K1 revertant plant, one reverted back to a *svt2*-like phenotype ( Table 3), i.e., the K1 double revertant switched from *svt2* phenotype in the M _1_ generation to a Col-like phenotype in the M _2_ generation, and then reverted back to a *svt2*-like phenotype in the M _3_ generation. Note that only a small number of progeny was planted. In a second experiment, the *svt2* Col R1 revertant produced 20 individuals displaying a *svt2*-like phenotype ( Table 3). This represents a larger reversion percentage than in the K1 double revertant (22.7% vs. 1.6%). This may be explained by the genotypic make-up of the Col-like reverted parents and will be presented in the next section. Figure 8 illustrates the phenotypic appearance of three examples of *svt2* → Col single revertants (Col R1, Col R2, K1 Col R) and a *svt2* → Col → *svt2* double revertant (K1 Col R *svt2* R).

**Figure 9.**
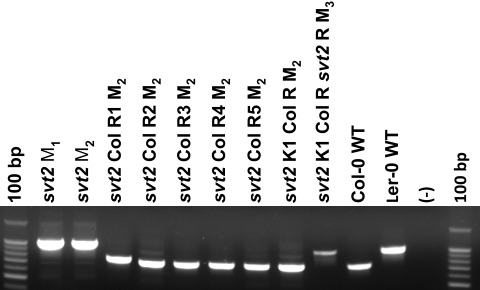
Insertion/Deletion polymorphism analysis in *svt2*, Col-0, L *er*-0, and revertants. PCR amplification of the Col/Ler *VTC1* promoter polymorphism in *svt2* plants and *svt2* revertant (R) plants, amplified with the *VTC1* G1F and G2R primers. (-) indicates negative control, no DNA.

Next we tested whether a Col-like revertant phenotype correlated with a Col-like genotype. Likewise, we would expect that a *svt2* → Col → *svt2* double revertant phenotype corresponds with *svt2*-like genomic markers. To check this we isolated genomic DNA from Col-0 and L *er*-0 wild types, *svt2*, *vtc1-1* and revertant mutants, and PCR-amplified the five randomly selected InDel polymorphisms plus the InDel polymorphism in the *VTC1* promoter ( Table 1). In all cases but the *svt2* M _2_ Col R1 revertant, the *svt2*-like revertant plants (labeled *svt2* M _2_ Col revertants 1 through 5) produced PCR products that where of the same electrophoretic mobility as the PCR products generated using Col-0 wild-type genomic DNA. In contrast, *svt2* M _1_ plants and *svt2* M _2_ plants that displayed an *svt2* phenotype, gave rise to PCR products that were of the same electrophoretic mobility as those of the L *er* wild type ( Table 4, Figure 9). In addition, the double revertant plant K1 (labeled *svt2* M _2_ K1 Col R) was genotyped in both its M _2_ and M _3_ generations. The K1 plant produced InDel PCR products similar to those of the Col-0 wild type in the M _2_ generation. However, the M _3_ generation that displayed *svt2*-like morphology produced PCR products that were comparable to the InDel PCR products generated using L *er* genomic DNA ( Table 4). The *svt2* M _2_ Col R1 (highlighted in red in Table 4 is intriguing, because it appears to contain DNA that is similar to both Col and L *er* genomic DNA. This suggests the presence of chimeric genome sectors, which may explain the higher percentage of Col-like revertants compared to *svt2* M _2_ K1 Col R. Note that the PCR results are in line with the sequencing analysis of the revertants. That is, Col-like revertants and *svt2*-like revertants share sequence similarity with Col-0 and L *er* wild type, respectively ( Figure S2).

**Table 4.** Summary of PCR-based molecular genotypes. With the exception of *svt2* Col R1 M _2_, where Col and L *er* markers and one heterozygous marker were found (highlighted in red), phenotype matched genotype. That is, a Col-like phenotype correlated with the presence of Col polymorphisms, while a L *er*-like phenotype correlated with L *er* polymorphisms. C, L, and H refer to Col, L *er*, or heterozygous, respectively. R denotes revertant. n.d., not detected.

Genotype	InDel 1 450919	InDel 2 451470	InDel 3 469762	InDel 4 449053	InDel 5 455100	G1F + G2R *VTC1*
Col-0 WT	C	C	C	C	C	C
*vtc1-1*	C	C	C	C	C	C
L *er*-0 WT	L	L	L	L	L	L
*svt2* M _1_	L	L	L	L	L	L
*svt2* M _2_	L	L	L	L	L	L
*svt2* Col R1 M _2_	C	L	C	H	C	C
*svt2* Col R2 M _2_	C	C	C	C	C	C
*svt2* Col R3 M _2_	C	C	C	C	n.d.	C
*svt2* Col R4 M _2_	C	C	C	C	C	C
*svt2* Col R5 M _2_	C	C	C	C	C	C
*svt2* K1 Col R M _2_	C	C	C	C	C	C
*svt2* K1 Col R *svt2* R M _3_	L	L	L	L	L	L

Summary of PCR-based molecular genotypes With the exception of svt2 Col R1 M2, where Col and Ler markers and one heterozygous marker were found (svt2 M2 Col-0 rev.1), phenotype matched genotype. That is, a Col-like phenotype correlated with the presence of Col polymorphisms, while a Ler-like phenotype correlated with Ler polymorphisms. C, L, and H refer to Col, Ler, or heterozygous, respectively

Summary of PCR-based molecular genotypes Table 4 raw data gel images

Taken together, these data suggest (i) transgenerational phenotypic and genotypic instability in *svt2*, and that (ii) *svt2* offspring do not segregate in a Mendelian fashion. In an attempt to obtain first insights toward a mechanism that is causing this genotypic instability, we investigated whether transgenerational epigenetic inheritance could play a role.

### Genome instability in *svt2* does not appear to be triggered by a transgenerational epigenetic mechanism

To investigate whether genome instability is caused by transgenerational epigenetic inheritance in the *svt2* mutant, we performed reciprocal crosses between *svt2* mutants and Col-0 wild-type plants. It is possible that through the EMS mutagenesis of *vtc1-1* seeds, genes involved in the regulation of epigenetic alterations were altered, whereby their activity was affected. There is increasing evidence in both plants and animals that epigenetic marks are not always cleared between generations. Incomplete erasure at genes associated with a measurable phenotype results in unusual patterns of inheritance from one generation to the next, termed transgenerational epigenetic inheritance ^22, 23^. Therefore, analysis of the progeny of the reciprocal crosses is expected to provide some first insights on the possibility of transgenerational epigenetic inheritance that is transmitted maternally. If this were the case, only progeny of crosses with a maternal *svt2* donor should have a *svt2*-like phenotype. To determine the genotypes of the F _1_ progeny of the reciprocal crosses, we performed another InDel polymorphism assay as described above. In addition, progeny were also screened using the *VTC1* InDel promoter polymorphism. Table 5 contains a summary of the InDel screen for progeny from each reciprocal cross. In all but six of the progeny from the reciprocal crosses, PCR products similar to those obtained using Col and L *er* genomic DNA, respectively, were generated, suggesting that the F _1_ of the reciprocal crosses were heterozygous. A similar result was obtained for the *VTC1* promoter polymorphism marker in all reciprocal crosses. Note, however, that for some polymorphisms and irrespective of whether *svt2* or Col-0 served as female or male donor, respectively, PCR products comparable to those obtained using L *er*-0 wild-type DNA were prevalent (highlighted in red in Table 5). This is surprising because heterozygosity was expected at all loci. This suggests that some parts of the genome were not inherited equally from both parents. Taken together, these results suggest that maternal epigenetic inheritance may not be the cause of genome instability in *svt2*. However, at some loci *svt2*-like alleles dominate over Col-0.

**Table 5.** Reciprocal crosses between *svt2* and Col-0 wild-type lines. Molecular analysis of the InDel polymorphism markers showed evidence of cryptic but persistent homozygosity, irrespective of the direction of the sexual cross (highlighted in red). However, heterozygosity was expected at all loci.

Female × Male crosses	InDel 1 450919	InDel 2 451470	InDel 3 469762	InDel 4 449053	InDel 5 455100	G1F + G2R *VTC1*
*svt2* × Col-0 F _1_ 1	H	H	L	H	H	H
*svt2* × Col-0 F _1_ 2	H	H	L	H	H	H
*svt2* × Col-0 F _1_ 3	H	H	H	H	H	H
*svt2* × Col-0 F _1_ 4	H	H	H	H	H	H
Col-0 × *svt2* F _1_ 1	H	H	H	L	H	H
Col-0 × *svt2* F _1_ 2	H	H	L	H	H	H
Col-0 × *svt2* F _1_ 3	H	H	H	L	H	H
Col-0 × *svt2* F _1_ 4	H	H	L	H	H	H

Reciprocal crosses between svt2 and Col-0 wild-type lines Molecular analysis of the InDel polymorphism markers showed evidence of cryptic but persistent homozygosity, irrespective of the direction of the sexual cross (L1). However, heterozygosity was expected at all loci. n.d., not detected. * indicates that a PCR product failed to generate for these reactions

Repeated PCR reactions of reciprocal crosses between svt2 and Col-0 wild-type lines Numbers correspond to gel lanes above. Letters C and H indicate Col or heterozygous genotype, respectively. n.d., not detected

Reciprocal crosses between svt2 and Col-0 wild-type lines Table 5 raw data gel images

Repeated reciprocal crosses between svt2 and Col-0 wild-type lines Table 5 raw data gel images (repeats)

## Discussion

The *svt2* mutant was initially identified as a putative suppressor of the AA-deficient *Arabidopsis* mutant *vtc1-1*, as was evident in wild-type levels of AA ( Figure 3C) and recovered root development in the presence of ammonium ( Figure 3D). However, *svt2* manifests other interesting characteristics, including genotypic and phenotypic instability. These unique features could aid in our understanding of the complex mechanisms controlling genome instability and restoration.

### *svt2* is a novel *Arabidopsis* mutant and not a result of an experimental artifact, seed contamination, or outcrossing

Several lines of evidence support our findings that *svt2* is a novel mutant. First, *svt2* was the only suppressor mutant isolated among over 1000 EMS-mutagenized M _0_ seeds to show unique phenotypic characteristics. Astonishingly, our genetic analysis revealed that both maternal and paternal alleles were affected in five randomly selected InDel polymporphism loci, the newly discovered InDel polymporphism in the *VTC1* promoter, and additional SNPs ( Figure 5B–D, Figure 6, Figure S1). These data demonstrate that *svt2* has acquired new characteristics, presumably as a result of EMS mutagenesis, and that *svt2* is neither Col nor L *er*. These data also argue against *svt2* being an experimental or PCR artifact.

Second, a number of data provide strong arguments against seed contamination. (1) With high reproducibility, descendents of the original *svt2* mutant produce offspring revertants with Col-like features ( Table 3, Table 4; Figure 8, Figure 9). (2) One of the Col-like revertants, *svt2* Col R1 M _3_, exhibited heterozygosity at some of the InDels tested ( Table 4). (3) One of those Col-like revertants, *svt2* K1 Col R M _3_, produced progeny that reverted back to *svt2*-like plants ( Table 3, Table 4; Figure 8, Figure 9). (4) We were unable to obtain true F _1_ heterozygotes in all *svt2*/Col-0 reciprocal crosses ( Table 5). (5) Delayed flowering and enlarged morphology phenotypes argue against the fact that *svt2* is a result of a L *er*-0 wild-type seed landing on the flat during the initial planting of the *vtc1-1* M _0_ mutagenized population. There is the possibility of a L *er* seed contamination of the *vtc1-1* seed stock used for EMS mutagenesis. Although we have sequenced the *vtc1-1* seed stock used for this experiment and confirmed that it is homozygous for the *vtc1-1* mutation, one could argue that sequencing the seed stock may not be a sensitive enough method to rule out contamination with a few L *er* seed. We performed many other experiments using this very same seed stock and never observed L *er*-like plants among the *vtc1* population. However, arguments (1) through (4) above speak most compellingly against seed contamination.

Third, the following experimental evidence argues against the possibility that *svt2* was generated by cross pollination of *vtc1-1* mutant plants with L *er* wild-type plants. (1) If *svt2* were generated by L *er* cross-pollination, the InDel polymorphism markers tested using *svt2* genomic DNA should have indicated heterozygosity. This, however, was not the case ( Table 4). (2) While *svt2* shares phenotypic and genotypic characteristics with L *er* and Col, it also has unique features ( Figure 3A, Figure S1). (3) *svt2* exhibits phenotypic and genotypic instability, causing the appearance of revertants with persistent reproducibility. (4) L *er* plants were not grown in our growth chambers at the time of the mutagenesis experiment. Furthermore, *svt2* was isolated by placing Aracons over the mutant plant to allow self-fertilization and seed production.

### Possible causes of genome instability in *svt2*

Our results are indicative of genome instability in *svt2*. Genome instability may be a result of polyploidy ^24^. Polyploids can arise from genome duplication (autopolyploids) or interspecific hybridization (allopolyploids). Our data suggest that *svt2* does not contain multiple sets of chromosomes, because *VTC1* occurs as a single copy gene in *svt2* and *vtc1-1* mutants as well as the Col-0 and L *er*-0 wild-type controls ( Table 2). Furthermore, extra DNA must be replicated with each cell division. Therefore, enlarged cell size is often associated with polyploids ^25^. The chemical mutagenesis of *vtc1-1* seed could have resulted in mutations, which may have led to increased ploidy levels in one, two, or all three meristem layers, L1, L2, and L3. However, only mutations in the L2 layer, which gives rise to the reproductive organs, are inherited. Polyploidy in the L2 layer is reflected in pollen size. While *svt2* has an overall enlarged morphology ( Figure 3A), its pollen size is comparable to that of the other three genotypes ( Figure 4). This suggests that *svt2* anthers are not polyploid. Finally, allopolyploids often display a greater degree of heterozygosity ^25^, low fertility, and low embryonic viability ^26– 28^. This, however, is not the case in *svt2*. The fact that *svt2* is fertile and that its enlarged morphology is heritable from one generation to the next suggests that *svt2* is neither a somatic nor a gametic polyploid. Thus, it is therefore unlikely that polyploidy in *svt2* contributes to genome instability. This is supported by Ruffio-Chable and co-workers, who reported that between 5% and 21% of F _1_ hybrids in *Brassica oleracea* showed aberrant leaf phenotypes, despite normal ploidy levels ^29^.

Instead, we hypothesize that genome instability of *svt2* was further aggravated by exposing the already instable genome of *vtc1-1* mutants to EMS. It has recently been shown that plants impaired in certain aspects of protection against reactive oxygen species have a higher incidence of spontaneous double-strand breaks ^30^. The AA-deficient *vtc1-1* mutant has a three-fold higher spontaneous homologous recombination frequency and has a higher incidence of double-strand breaks (see below). Similar results were reported for the *Arabidopsis thaliana* flavonoid-deficient mutants *tt4* and *tt5* ^30^. One may speculate that through the high level of stress induced by EMS, a yet unknown mechanism of genome restoration was turned on. In fact, genome alterations in soybean and flax in response to environmental stress have been reported previously ^31, 32^. In the process of soybean cell culture, massive specific changes in numerous genome-wide loci were observed ^31^. It was suggested that this genetic variation is a consequence of specific recombinational events. Similarly, in flax a single-copy 5.7 kilobase DNA fragment that was not present in the parent line appeared in genotrophs in response to particular growth conditions ^32^.

### Possible mechanisms of genome restoration in *svt2*

The experimental evidence described in this work raises the question as to what mechanism is responsible for the loss or reintroduction of genomic DNA sequences in the original *svt2* mutant and its revertant offspring. Several mechanisms may be considered: activity of transposable elements, random mutations, unequal crossing over, gene conversion, double-strand breaks and recombination, and activity of an RNA cache.

Transposons are DNA elements capable of moving around the genome; movement is often associated with chromosome breaks and formation of unstable mutations, which revert frequently but often give rise to new phenotypes. Movement of transposable elements often occurs during meiosis and mitosis and is accelerated by genome damage ^33^. These represent conditions that are present in *svt2*. However, transposons have a variety of molecular features that do not apply to *svt2*. Transposons exist as multiple copies in the genome. A blast search of the *VTC1* promoter insertion in *svt2* did not return any other hits, indicating that the DNA sequence is not present in its entirety anywhere else in the genome. Additionally, transposon termini represent inverted repeats. This, however, is not the case in *svt2* ( Figure S1). A short, direct repeat of genomic DNA often flanks the transposon, leaving a “footprint”. Our sequencing analysis of the *VTC1* promoter region in *svt2* did not reveal any footprints, suggesting that transposon activity is not responsible for the insertion or loss of novel sequences in *svt2* ( Figure S1).

Random mutations caused by EMS mutagenesis could have activated an unknown mechanism in *vtc1-1* seeds, giving rise to the phenome and genome instability in *svt2*. This may explain the novel SNPs we detected in *svt2* that are distinct from the *vtc1-1* mutant and Col-0 and L *er*-0 wild types ( Figure S1). The disappearance of the *vtc1-1* mutation in *svt2* ( Figure 5D, Figure S1) may also be explained by the introduction of a random mutation. However, it is possible that exposure of *vtc1-1* seeds to EMS could have reversed the original *vtc1-1* mutation to the wild-type sequence, as *vtc1-1* was initially isolated in an EMS screen ^15^. Interestingly, Conklin and co-workers previously isolated two *vtc1* alleles, *vtc1-1* and *vtc1-2*, containing the exact same single cytosine to thymine point mutation at amino acid position 64 relative to the start codon, despite the fact that *vtc1-1* and *vtc1-2* mutants were isolated independently from different EMS-mutagenized pools ^15^. The authors suggested that a limited number of mutations are tolerable in the VTC1 enzyme GDP- d-mannose pyrophosphorylase without causing embryo lethality. This is supported by the fact that several independently isolated *cyt* mutant alleles containing different amino acid mutations in *VTC1* are embryo lethal ^34^. To date, only the *vtc1-1* ^15^ and *hsn1* mutations ^8^ have been isolated and reportedly do not cause embryo lethality. This suggests some form of allelic constraint that has been reported in *Arabidopsis* previously ^35, 36^. Furthermore, in the EMS screen in which the *svt2* mutant was isolated, several other *vtc1-1* suppressor mutants with restored root development in the presence of ammonium were identified. Sequencing analysis revealed that in all of these mutants the *vtc1-1* mutation was restored to the wild-type allele, while the suppressor mutants neither exhibited a *svt2*-like phenotype nor did they produce revertants in the subsequent generation (Kempinski *et al.,* unpublished data).

Exposure to EMS or γ–radiation has been reported to induce high frequency phenotypic instability in the *Arabidopsis* disease resistance genes *CPR1* and *BAL*, which map to the *RPP5* locus ^16^. Yi and Richards reported destabilization of phenotypes in both the *bal* and *cpr1* mutants in more than 10% of EMS-treated plants in the M _1_ generation. They also identified exceptions to simple Mendelian inheritance in the M _2_ generation. Phenotypic instability was also observed in *bal* × *cpr1* F _1_ hybrids. The authors suggested that the high degree of phenotypic instability in *bal* and *cpr1* mutants is due to the fact that the *RPR5* locus can adopt different metastable genetic or epigenetic states, whose stability is highly susceptible to mutagenesis and pairing of different alleles. Yi and Richards later reported that the phenotypic instability of *bal* mutants is caused mainly by gene duplication and hypermutation of the *SNC1* gene ^17^.

As observed in the *cpr1* and *bal* mutants, we hypothesize that EMS treatment has destabilized the genome of *svt2* by interrupting one or more mechanisms involved in genomic inheritance. A combination of unequal crossing over, gene conversion, double-strand breaks, DNA recombination, and/or the presence of an RNA cache template may explain the loss and reappearance of DNA sequences in *svt2*. Genome-wide non-Medelian inheritance of extra-genomic information in *Arabidopsis* was reported in the *hothead* ( *hth*) *Arabidopsis* mutant ^37^. Self-fertilization of homozygous mutant plants resulted in approximately 10% *hth* revertants, which were *hth/HTH* heterozygous, suggesting that the *HTH* gene was altered in the progeny. However, the authors also detected rare homozygous revertants *HTH/HTH* embryos, which must have inherited one of their two wild-type *HTH* genes from the maternal parent and could not have been a result of outcrossing. Inheritable genome-wide high-frequency gene homozygosity in early generations in rice has also been reported ^38^. Lolle *et al.* postulated that these genetic restoration events are the result of a template-directed process that utilizes an ancestral RNA-sequence cache ^37^. This hypothesis is supported by observations reported by Xu and co-workers ^38^. Therefore, our genetic and phenotypic *svt2* data, in conjunction with the observed higher occurrence of double-strand breaks and spontaneous homologous recombination frequency in *vtc1-1*, are in support of the RNA cache theory. Additional studies are needed to provide experimental support for this hypothesis.

## Conclusions

We have isolated a novel *Arabidopsis* mutant that is capable of restoring genetic information that was not present in the chromosomal genome of its parents. We suggest that this ancestral information is present in some cryptic form that is accessible under extreme stress conditions. Genome restoration could be advantageous to plants that encounter environmental changes for which ancestral genes were better adapted. However, the mechanisms responsible for triggering and executing genome restoration remain to be determined. Double strand breaks, DNA recombination, and/or the activity of an RNA cache may be contributing factors. In the future, *svt2* may serve as a model to study non-Mendelian inheritance and could provide insight into the evolution and diversification of *Arabidopsis* ecotypes.

## Abbreviations

AA, ascorbic acid; EMS, ethyl methanesulfonate; InDel, Insertion/Deletion; MS, Murashige and Skoog.

## Figures and Tables

**Figure:**
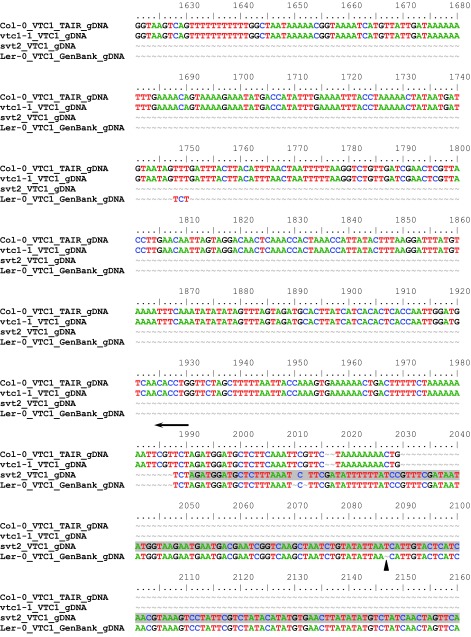

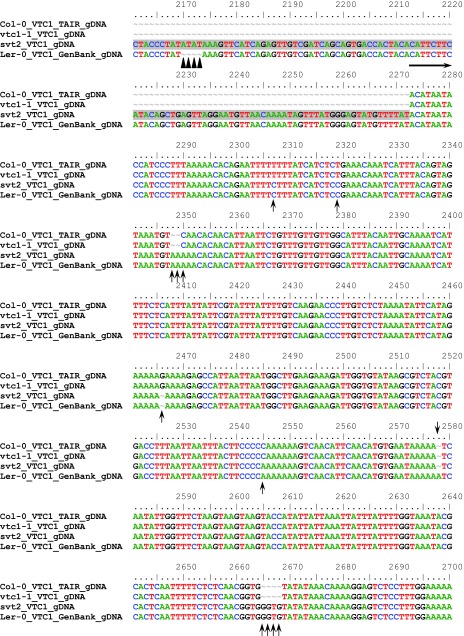

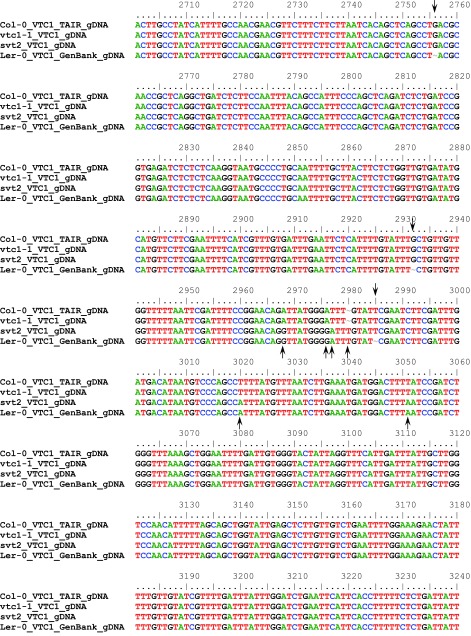

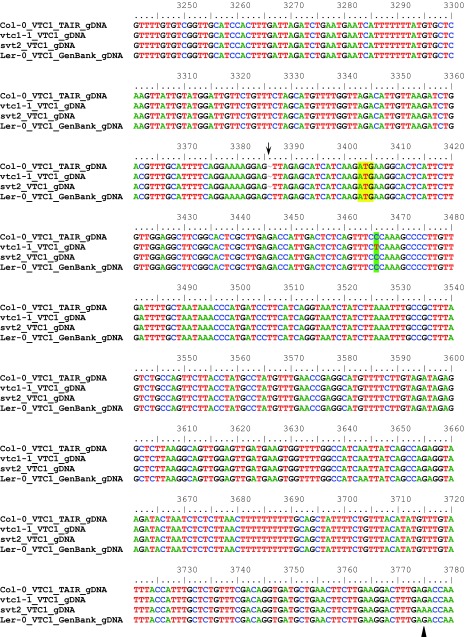

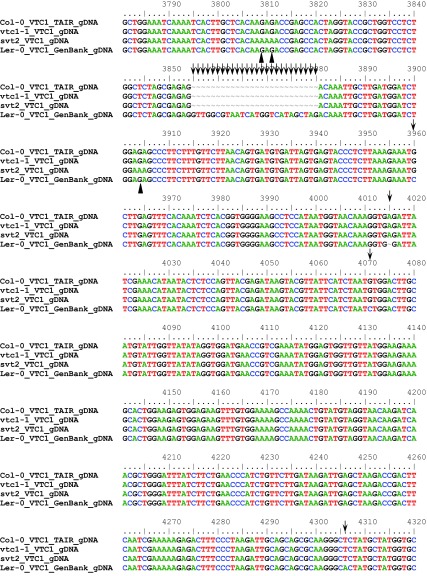

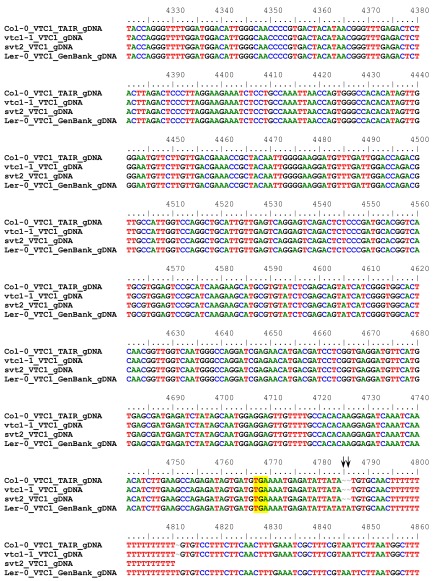


**Figure:**
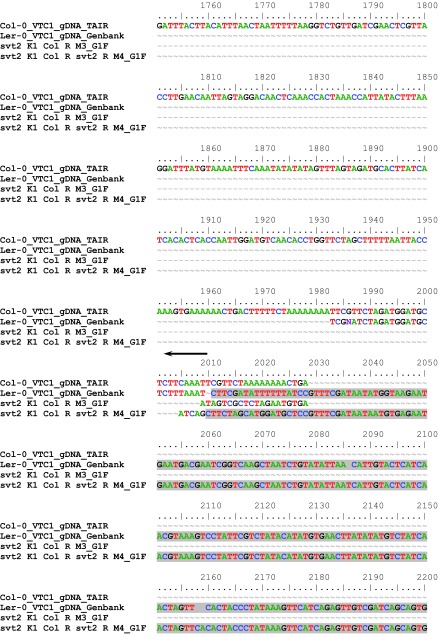

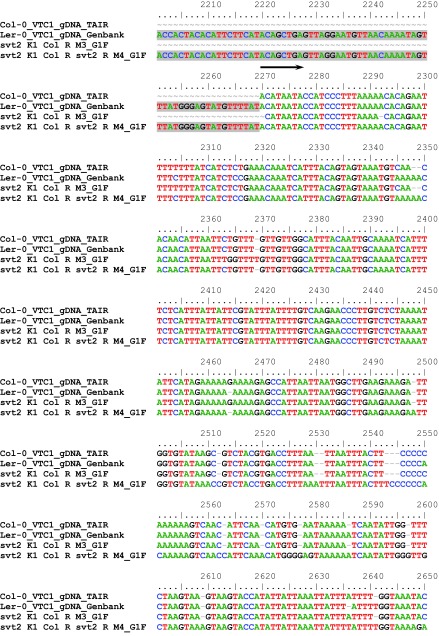

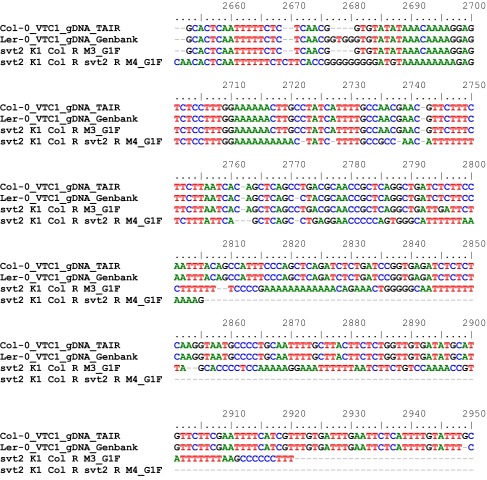


## Supplementary material

**Figure S1**. Sequence alignment of the *VTC1* gene sequence of the Col-0 TAIR database, the *vtc1-1*, *svt2* mutants, and the L *er*-0 GenBank database.

*Horizontal arrows denote 5´ respectively 3´ flanking regions of the sequence insertion, which is highlighted in grey, in the VTC1 promoter region (between base pairs 1990 and 2273). Upright arrows indicate sequences shared between *svt2* and L *er*. Arrows pointing down denote sequences shared between *svt2* and Col. Arrowheads point to sequences unique to *svt2*. Highlighted in yellow are the start and stop codons, respectively. Highlighted in green is the *vtc1-1* mutation.*

**Figure S2**. Sequence alignment of the *VTC1* promoter InDel polymorphism sequence of the Col-0 TAIR database, the L *er*-0 Genbank database, the *svt2* K1 Col R M _3_ revertant (Col-like phenotype) and the *svt2* K1 Col R *svt2* R M _4_ revertant ( *svt2*-like phenotype).

*Horizontal arrows denote 5´ respectively 3´ flanking regions of the sequence insertion, which is highlighted in grey, in the *VTC1* promoter region in L *er*-0 and *svt2* K1 Col R *svt2* R M4, which exhibits an *svt2*-like phenotype. The *svt2* K1 Col R M3 mutant has a Col-like phenotype and share sequence similarities with the Col-0 sequence. R denotes revertant.*
