# Supplementary figures and images for: The novel Arabidopsis thaliana svt2 suppressor of the ascorbic acid-deficient mutant vtc1-1 exhibits phenotypic and genotypic instability
Source: F1000Res. 2013 Jan 10;2:6. [Version 1] doi: 10.12688/f1000research.2-6.v1 (PMC3938180; doi:10.12688/f1000research.2-6.v1)

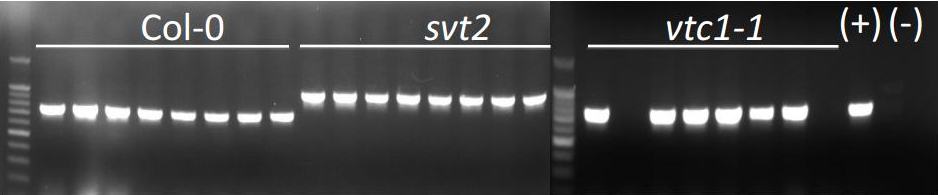

Supplement: PCR amplification of the VTC1 promoter region in the Col-0 wild type, vtc1-1 and svt2 mutants — Additional raw data of PCR amplification of the VTC1 promoter region using the VTC1 G1F and VTC1 G2R primers in genomic DNA isolated from individual Col-0 wild type, svt2, and vtc1-1 plants (N=8 for each). Positive and negative controls are indicated as (+) and (-), respectively. [file f1000research-2-866-s0007.tgz › Figure_5B_raw_data_VTC1_G1F_and_VTC1_G2R.jpg]

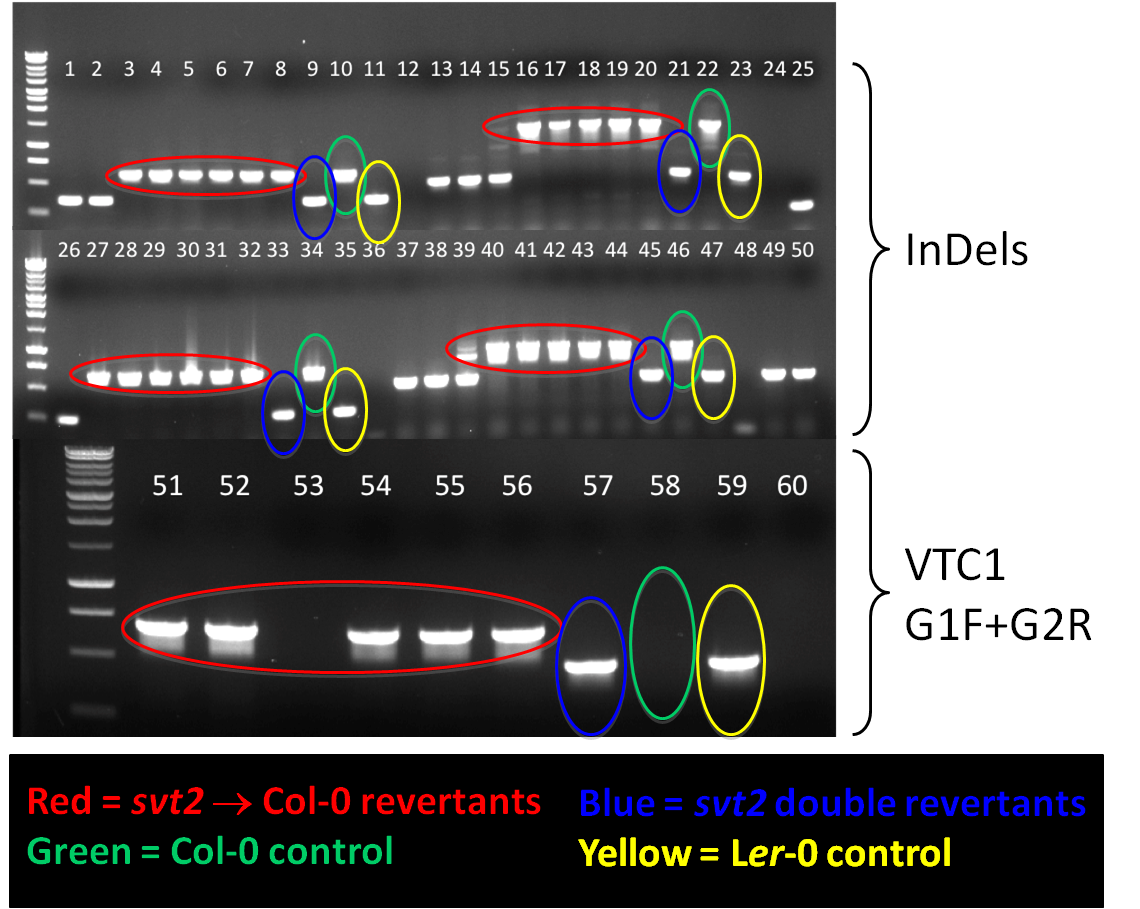

Supplement: Summary of PCR-based molecular genotypes — Table 4 raw data gel images [file f1000research-2-866-s0010.tgz › Table_4_raw_data_PCR_based_molecular_genotypes_image.png]

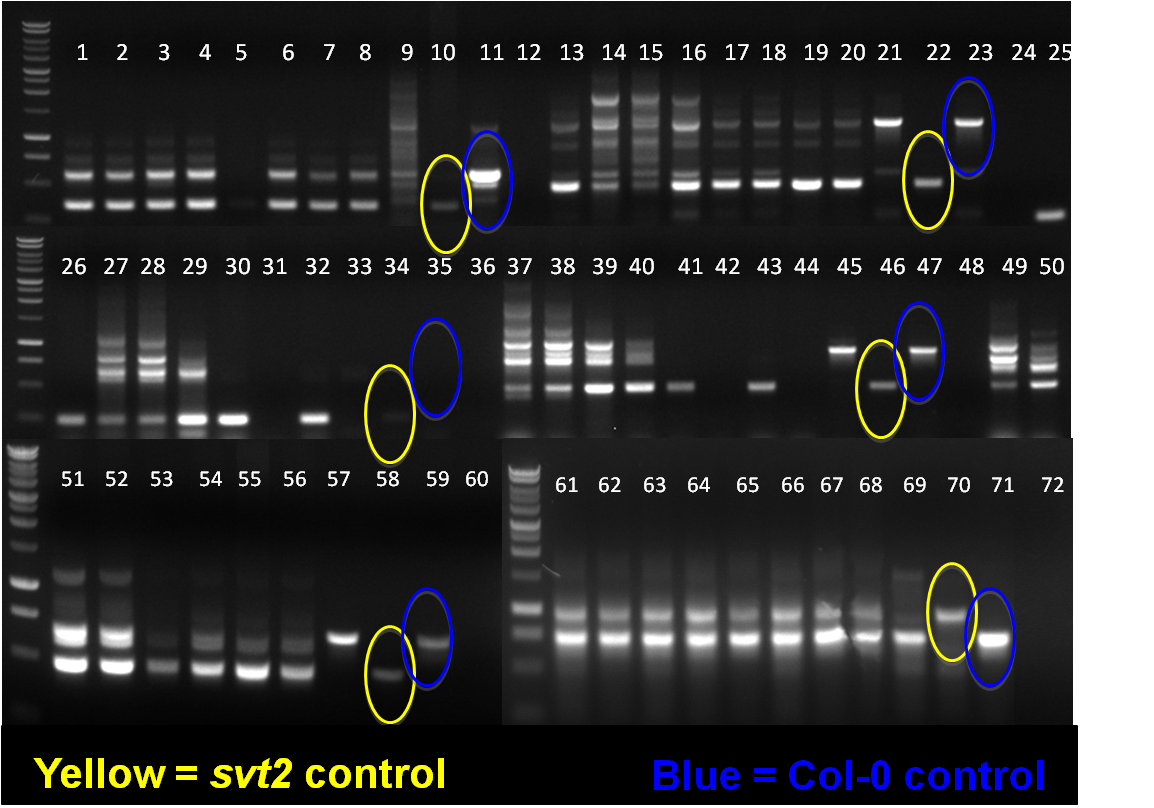

Supplement: Reciprocal crosses between svt2 and Col-0 wild-type lines — Table 5 raw data gel images [file f1000research-2-866-s0000.tgz › Table_5_raw_data_svt2_Col_WT_crosses_image.png]

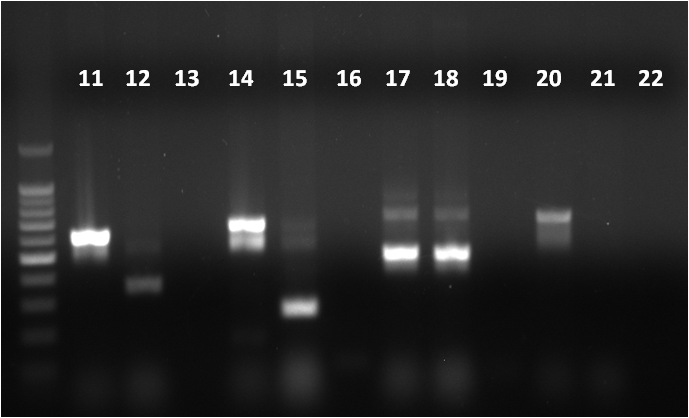

Supplement: Repeated reciprocal crosses between svt2 and Col-0 wild-type lines — Table 5 raw data gel images (repeats) [file f1000research-2-866-s0001.tgz › Table_5_raw_data_svt2_Col_WT_repeated_crosses_image.png]
