# Supplementary material for: The novel Arabidopsis thaliana svt2 suppressor of the ascorbic acid-deficient mutant vtc1-1 exhibits phenotypic and genotypic instability
Source: F1000Res. 2013 Jan 10;2:6. [Version 1] doi: 10.12688/f1000research.2-6.v1 (PMC3938180; doi:10.12688/f1000research.2-6.v1)
Supplement: Peer review history

# Peer review: Referee response for version 1

**Published:** 2013-01-31

**Authors:** David Oppenheimer

**Affiliations:** 1 Department of Botany, University of Florida, Gainesville, FL, USA

In this paper, the authors characterize a suppressor of the *vtc1-1* mutation, which they named *svt2*. The authors characterize the phenotype of the *vtc1-1* mutants that carry the suppressor mutation and show that the plants have characteristics reminiscent of the L *er* accession. Molecular characterization of the suppressed plants show that the suppressor mutation is apparently a reversion of the original *vtc1-1* mutation, and surprisingly, have additional genomic signatures of the L *er* ecotype as well as additional mutations.  There are several problems with the experimental methods used in this manuscript. First, according to Figure 1 of the manuscript, the authors screened the M _0_ generation (the mutagenized seed) for wt ascorbic acid content. This is a significant problem for the subsequent mutant analysis in this manuscript. When *Arabidopsis* seed are mutagenized, the individual cells of the meristem on the seed are mutagenized independently. When the seed germinate, the plants are genetic mosaics. In addition, only those mutations in the L2 layer that gives rise to the germ cells will pass on the mutations. Therefore, it is highly unlikely that a seedling with wt ascorbic acid levels would be isolated from the M _0_, because it would take the accumulation of many independent mutations, each of which would need to lead to suppression of *vtc1. * It is possible that a large sector of an M _0_ seedling could contain a suppressor mutation that leads to wt ascorbic acid levels, but this sector would have to include cells in the L2 layer for the mutation to be passed on to the next generation. Also, an L *er*-like sector should be obvious on a mostly Col-0 plant. Nonetheless, a sector that included the L2 would lead to segregation of the phenotype in the M _1_ generation, because the cells in the sector would be heterozygous for the suppressor mutation.  Second, the concentration of EMS commonly used for mutagenesis in *Arabidopsis* (0.2%) is known to cause multiple mutations per genome. When one isolates a mutant of interest from an EMS screen, one should back-cross it at least once to allow these other mutations to segregate away. Otherwise, one may observe unexpected results when analyzing the mutant of interest due to the effects of these other mutations.  Third, when analyzing the sequence of the *vtc1* gene in the original mutant and in the suppressor, *svt2*, the authors compared the sequence to the L *er* and Col-0 sequences reported in Genbank and TAIR. Instead, the authors should sequence the *vtc1* gene from their original *vtc1-1* stock and the L *er* accession that is present in their lab. This is because it is known that nucleotide polymorphisms arise regularly in lab stocks such that a comparison between a lab stock of Col-0 and the reference sequence can show many differences. Because the authors are reporting unexpected sequencing results, they should show the actual sequence traces (from both strands) for the individual base pair differences highlighted in Figure S1. It would be appropriate  to show these sequence traces in the supplemental data. Showing the sequencing traces for the base pair differences would demonstrate that the sequence differences are not due to ambiguous base calling or other sequencing errors.  Fourth, the authors refer to the suppressed plants and their revertants has having a L *er*-like or Col-like phenotypes. Because the phenotype of *vtc1* is lower ascorbic acid levels, and the putative suppressor has wt ascorbic acid levels, the ascorbic acid levels in the revertants should be measured to show that they are revertants, instead of relying on the L *er* or Col phenotype. Fifth, because seed and pollen contamination can explain the results, the authors need to explicitly state the degrees to which they tried to eliminate these possible sources of contamination. Were plants of more than 1 genotype grown together? Were seeds of more than 1 genotype collected in the same room? Was soil stored where plants were setting seed? etc.  The single, L *er-*like seedling found in the M _0_ population can be explained as an L *er* seed that contaminated the Col-0 *vtc1-1* seed stock used for the mutagenesis. This can be tested by sowing several thousand Col-0 *vtc1-1* seed from that seed stock, and screening them for the presence of any L *er* contaminants.  The results in Table 4 are the same as one would expect from seed contamination: the *svt2* plants (with the L *er* phenotype) have all L *er *markers, and the revertants (with the Col phenotype) have all Col markers. The *svt2* Col R1 M _2 _plant highlighted in red shows the expected results if the parent of that plant was heterozygous for Col/L *er*. Again, the authors should state what extraordinary measures they used to eliminate seed and pollen contamination.  Once these comments are addressed, the other unexpected results can be examined in a new light.

I have read this submission. I believe that I have an appropriate level of expertise to state that I do not consider it to be of an acceptable scientific standard, for reasons outlined above.

# Peer review: Referee response for version 1

**Published:** 2013-01-31

**Authors:** Igor Kovalchuk

**Affiliations:** 1 University of Lethbridge, Lethbridge, AB, Canada

Very unusual story. I am still puzzled how this is all possible. I can assume that original vtc1 line had some Ler-1 background (may be from backcrosses). In this case it is possible that the seeds you started with for mutagenesis are highly heterogeneous and some have Ler genomes still present. Now, such a severe case of rearrangements due to combination of EMS and vtc1 background is unbelievable. I wonder why other plants with even greater instability, such as ddm1 or msh2, have never had anything like this reported. Maybe they have not looked for it hard enough? It would make sense to get the vtc1 mutant into rdr2 or rdr6 background (or both) and see whether this RNA cache plays any role - I would expect much lower chance of getting those revertants, same with reverse transcription mutants. 

I understand that the event is rare – a single plant was produced – but it would really make the entire story stronger if several different plants were produced.

I have read this submission. I believe that I have an appropriate level of expertise to confirm that it is of an acceptable scientific standard.

# Peer review: Referee response for version 1

**Published:** 2013-01-25

**Authors:** Andy Pereira

**Affiliations:** 1 Departments of Crop, Soil and Environmental Sciences, and Plant Pathology, University of Arkansas, Fayetteville, AR, USA

Very interesting experimental evidence of an inheritance phenomenon that is non-Mendelian and supports an RNA cache hypothesis. The data support the conclusions drawn, but some alternative explanations are put forth that can be addressed.

The EMS mutagenesis treatment of the *vtc1-1* genotype yields a single suppressor *svt2* mutant that turns out to be a revertant of the *vtc1-1* mutation, and is homozygous. Since this screen was from ~1200 seed, it would be interesting to know if such revertant suppressor mutations are also be present in the original batch of *vtc1-1* seed used for mutagenesis. Of course since the screen entails a tedious test of TTB on leaves of individual plants for AA content, it is not a recommended control test that should be done, but mechanistically the question remains if the locus is mutable without mutagenesis. What is curious is that the phenotype of the suppressor plant shows a L *er* ‘plant type’ phenotype.  

Looking back at the history of the *vtc1* mutant, the only time when the L *er* and Col genomes were together, described in Conklin *et al *(1996), was when the *vtc1 *mutant was crossed to L *er* for mapping. The description of the *vtc1-1* ( *soz1*) mutant stock (in TAIR) is given as result of 2 x backcrosses and an F3, presumably as a result of crossing to Col-0, but it might be useful to confirm that the stock has no L *er* background and the seed used was progeny of single plant and not from a bulk seed lot. Since the *VTC1* locus has also been characterized by *cyt1* and *emb101* mutants, it would seem that the mutant alleles might have some disadvantage in being propagated and a ‘residual heterozygosity’ might persist by some mechanism. In addition, reversion to a wild-type phenotype *svt2* might be facilitated by a selection of vigorous embryos into maturity.The sequence changes in the *vtc1-1* and *svt2* suggests an origin of a ‘template’ independent of L *er* and Col alleles, and might also be sequences from another related ecotype. A screen of available Arabidopsis ecotype genome sequences should show such an alternate donor.

Minor comment: On the PDF, page 10, need to use ‘were’ instead of 'where' in the sentence beginning "In all cases..."

I have read this submission. I believe that I have an appropriate level of expertise to confirm that it is of an acceptable scientific standard.
